# Integrating MASLD detection into diabetes care in primary care settings in Mexico: A cascade analysis

**DOI:** 10.1016/j.clinme.2026.100585

**Published:** 2026-04-18

**Authors:** Rubén Silva-Tinoco, Erick Vladimir Martínez-de la Cruz, Ana Galíndez-Fuentes, Berenice Cabrera-Victoria, Erick Villa-Mejía, Ricardo Ulises Macías-Rodríguez, Alejandro Avalos-Bracho, María Fernanda Bernal-Ceballos

**Affiliations:** a Unidad de Atención a la Salud, Servicios Públicos de Salud del Instituto Mexicano del Seguro Social para el Bienestar, Mexico City, Mexico; b Clínica Especializada en el Manejo de la Diabetes en la Ciudad de México, Servicios Públicos de Salud del Instituto Mexicano del Seguro Social para el Bienestar, Mexico City, Mexico; c Servicio de Radiología Intervencionista, Centro Médico Nacional La Raza, Mexico City, Mexico; d Department of Gastroenterology and Liver Transplant, Instituto Nacional de Ciencias Médicas y Nutrición Salvador Zubirán, Mexico City, Mexico

**Keywords:** Metabolic dysfunction-associated steatotic liver disease, Liver fibrosis, Diabetes care, Primary care

## Abstract

**Background:**

Metabolic dysfunction-associated steatotic liver disease (MASLD) remains underrecognised in diabetes care due to the lack of structured detection pathways in primary healthcare.

**Objective:**

To evaluate the feasibility and performance of a stepwise diagnostic pathway for identifying the full spectrum of MASLD in adults with type 2 diabetes in primary healthcare settings.

**Methods:**

A diagnostic pathway for early detection across the MASLD spectrum, including steatosis, metabolic dysfunction-associated steatohepatitis (MASH), liver fibrosis risk and significant fibrosis, was integrated into a multicomponent diabetes care programme within the public health system. A secondary exploratory analysis used multivariable logistic regression to identify factors independently associated with MASLD.

**Results:**

Among 454 adults with type 2 diabetes, MASLD was identified in 51.5% and MASH in 22.2%. Through stepwise assessment, 22.7% showed intermediate-to-high fibrosis risk, and among those who underwent transient elastography, significant fibrosis was confirmed in 27.9% of individuals with increased risk, yielding an estimated prevalence of 10.6% in the MASLD group and 5.9% overall. In multivariable analysis, female sex, elevated liver enzymes and higher body mass index were independently associated with MASLD, with a non-linear association across BMI categories.

**Conclusion:**

Implementing structured care pathways for the identification and management of MASLD is feasible and essential to mitigate the burden of diabetes and liver disease. Strengthening primary care capacity remains crucial for systematic integration of MASLD identification in routine care.

## Highlights

• A MASLD diagnostic pathway was implemented in primary diabetes care.
• MASLD was identified in 51.5% of adults with type 2 diabetes.
• One in five patients met criteria for MASH or had fibrosis risk.
• FIB-4 identified 22.7% with intermediate-to-high fibrosis risk.
• Female sex, higher BMI and elevated ALT/AST were independently associated with MASLD.

## Graphical Abstract

**Figure:**
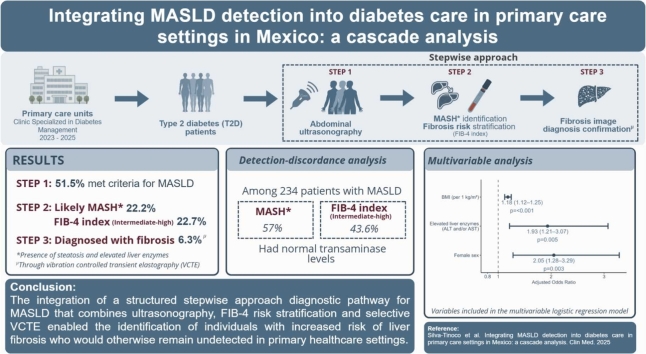

## Introduction

Diabetes is a major contributor to the global disease burden, largely due to its multisystem involvement, including hepatic complications.^1^ Metabolic dysfunction-associated steatotic liver disease (MASLD) encompasses a spectrum of liver conditions that can progress to fibrosis, cirrhosis and hepatocellular carcinoma. MASLD and diabetes are bidirectionally linked, with each condition exacerbating the other.^2^, ^3^ Furthermore, MASLD pathophysiology may accelerate extrahepatic complications like cardiovascular disease, worsening overall outcomes.

Despite its significant burden, MASLD remains underrecognised due to lack of systematic screening in diabetes care, particularly in primary care setting where guidelines recommend initial assessment should occur.^4^

Mexico faces a severe dual epidemic of diabetes and obesity, key drivers of MASLD.^5^, ^6^ With limited data to guide care improvements, this study implemented and evaluated a comprehensive MASLD diagnostic pathway aligned with international recommendations within Mexico’s public health system. Therefore, this study aimed to evaluate a comprehensive diagnostic pathway covering the full spectrum of MASLD stages, in accordance with current international recommendations, within Mexico’s public health system.

## Methods

This quality improvement project introduced a diagnostic pathway for MASLD as part of a multicomponent diabetes care programme within Mexico’s public primary healthcare system. One of the main components of this initiative is to accelerate the implementation of evidence-based interventions to improve the prognosis of people with diabetes in primary care, including the timely detection of diabetes-related complications.^7^

Adults with type 2 diabetes (T2D) were referred from primary care units to the Specialist Clinic in Diabetes Management in Mexico City and consecutively assessed between August 2023 and February 2025. A stepwise diagnostic pathway for MASLD was integrated into routine diabetes care according to current clinical guidelines.^8^, ^9^ In the first step, all participants underwent abdominal ultrasonography to detect hepatic steatosis, which was subsequently graded as mild (S1), moderate (S2) or severe (S3). Because the MASLD definition includes the presence of at least one cardiometabolic risk factor, participants with steatosis and T2D in this study were classified as having MASLD. Patients were identified with metabolic dysfunction-associated steatohepatitis (MASH) when steatosis coexisted with elevated liver enzymes (ALT >33 U/L in men and >25 U/L in women).^9^

In the second step, fibrosis risk was estimated using the FIB-4 index, calculated as age (years) × AST / [platelets (10^9^/L) × √ALT]. Cut-off points of <1.3, 1.3–2.67 and >2.67 defined low, intermediate and high fibrosis risk, respectively; for those who were aged >65 years, the FIB-4 cutoff for low risk was 2.0.^8^

In the third step, patients with intermediate or high FIB-4 index scores were referred to a tertiary care centre for secondary assessment for liver fibrosis by vibration-controlled transient elastography (VCTE). Liver stiffness measurement (LSM) ≥8.0 kPa was considered indicative of significant liver fibrosis (≥F2).^4^, ^8^ The diagnostic cascade quantified the proportion of patients identified at each stage of assessment.

## Statistical analysis

Continuous variables are presented as median (p25–p75), and categorical variables are expressed as absolute and relative frequencies. Differences were analysed using the Mann–Whitney *U* test, Pearson’s chi-square test and Fisher’s exact test for continuous and categorical variables, respectively.

We used logistic regression to examine associations with MASLD. Each candidate factor was first evaluated in a univariable logistic regression model, reporting odds ratios (ORs) with 95% confidence intervals (CIs). A multivariable model was then developed from a prespecified set of clinically plausible covariates; to obtain a parsimonious final model using backward elimination based on the Bayesian information criterion (BIC). To assess potential non-linearity of BMI, we additionally modelled BMI as a continuous variable using a spline-based logistic regression approach and estimated BMI-specific odds ratios for a 1 kg/m^2^ increase across the BMI, adjusted for the covariates retained in the final multivariable model. All tests were two-sided.

For variables with <10% missing data, we performed multiple imputation using random forest via the MICE algorithm using R version 3.5.3, generating and averaging five imputed datasets for final analysis. A significance level p < 0.05 was used.

A detection-discordance analysis compared the presence of steatosis or fibrosis risk between patients with and without elevated transaminases.

## Results

**Fig. 1:**
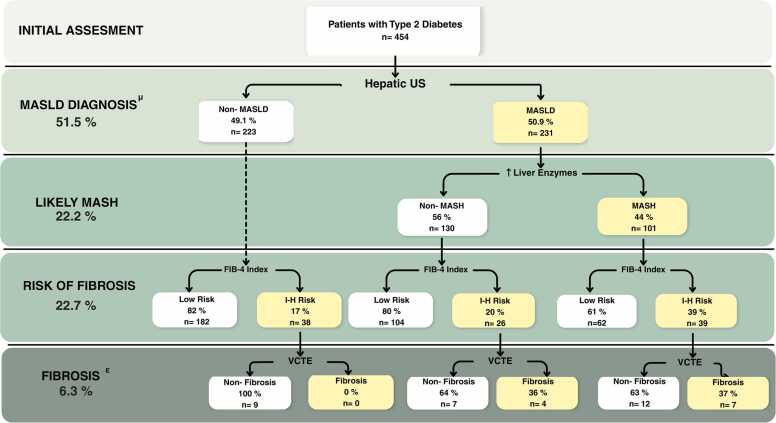
Diagnostic and fibrosis stratification cascade in patients with type 2 diabetes. Flowchart illustrating the sequential assessment of 454 patients with type 2 diabetes, including the diagnosis of metabolic dysfunction-associated steatotic liver disease (MASLD) by hepatic ultrasonography, the identification of metabolic dysfunction-associated steatohepatitis (MASH) based on liver enzyme elevation, fibrosis risk stratification using the FIB-4 index, and subsequent significant fibrosis confirmation with vibration-controlled transient elastography (VCTE). Percentages at each step indicate the proportion of patients meeting the corresponding criteria. I-H risk: Intermediate- and high-risk.^μ^ Patients detected with fibrosis downstream through VCTE (n = 3) were incorporated into the overall prevalence estimate. ^Ɛ^Adjusted based on patients with complete assessment using VCTE.

Of the 454 patients evaluated, steatosis was identified by ultrasonography in 231 (50.9%), establishing a diagnosis of MASLD. Three additional patients without ultrasonographic steatosis were subsequently reclassified as having MASLD after VCTE detected significant liver fibrosis. Consequently, the diagnostic pathway identified 234 patients (51.5%) with MASLD. The overall diagnostic cascade for MASLD in patients with T2D is presented in Fig. 1, illustrating each step of the detection and stratification process.

Characteristics of the 454 patients according to MASLD classification are shown in Table 1. The median age was 55 years (48–62) and 65% were female. Compared with non-MASLD group, those with MASLD had significantly higher BMI (30.6 vs 26.8 kg/m^2^; p < 0.001), waist circumference (105 vs 95 cm; p < 0.001), waist-to-hip ratio (0.96 vs 0.93; p < 0.001), liver enzymes (43.2% vs 30.9%; p = 0.001), serum creatinine (0.8 vs 0.7 mg/dL; p = 0.02), FIB-4 index (1.03 vs 0.91; p = 0.02), and lower HDL-c values (42.2 vs 45.4 mg/dL; p < 0.001). Among those identified with MASLD, the distribution of steatosis severity was as follows: S1, 81.6% (n = 191); S2, 16.6% (n = 39); and S3, 0.4% (n = 1).

**Table 1:** Characteristics of the study population stratified by the presence of MASLD.

Variable	Total n = 454	Non-MASLD n = 220	MASLD n = 234	p-value
Sex, female	294 (65)	147 (67.1)	147 (62.8)	0.40
Age, years	55 (48–62)	55 (48–64)	55 (48–62)	0.62
BMI, kg/m^2^	28.5 (25.5–31.6)	26.8 (24.2–29.2)	30.6 (27.6–33.3)	<0.001
Waist circumference, cm	100 (92–108)	95 (88–101)	105 (97–113.8)	<0.001
Waist-to-hip ratio	0.95 (0.90–0.99)	0.93 (0.90–0.97)	0.96 (0.92–1.01)	<0.001
**BMI categories**				
BMI malnutrition + normal, n (%)	97 (21.3)	66 (30)	31 (13.2)	<0.001
BMI overweight, n (%)	184 (40.5)	109 (49.5)	75 (32.1)	
BMI obese, n (%)	173 (38.1)	45 (20.5)	128 (54.7)	
Diabetes duration, years	10 (3–17)	10 (3–19)	10 (3–16)	0.08
Age at diagnosis, years	44 (36–52)	42 (34–51)	45 (37–52.7)	0.52
Age at diagnosis (<40 years), n (%)	163 (36)	88 (40.4)	75 (32)	0.06
Family history of DM, n (%)	39 (86.1)	187 (85)	204 (87.1)	0.57
Family history of CVD, n (%)	211 (46.4)	99 (45.2)	112 (48.4)	0.45
**Educational attainment, n (%)**				
Illiteracy	9 (1.9)	8 (3.6)	1 (0.4)	0.06
No education (can read and write)	19 (4.2)	11 (4.9)	8 (3.5)	
Primary	111 (24.5)	58 (26.6)	53 (22.6)	
Secondary	143 (31.6)	66 (30.2)	77 (32.9)	
High school	112 (24.7)	49 (22.1)	63 (27.3)	
University or more	57 (12.6)	25 (11.3)	32 (13.9)	
**Smoking status, n (%)**				
Current smoker	90 (19.8)	41 (18.8)	49 (20.9)	0.83
Former smoker	143 (31.4)	69 (31.6)	74 (31.6)	
**Alcohol consumption, n (%)**				
Never	152 (33.4)	68 (30.9)	84 (35.9)	0.67
Social	212 (46.6)	106 (48.2)	106 (45.3)	
Moderate-risk alcohol intake (*every 6 months or once a year*)	14 (3)	8 (3.6)	6 (2.6)	
High-risk alcohol intake (*daily to every 3 months*)	76 (16.7)	38 (17.3)	38 (16.2)	
**Comorbidities, n (%)**				
Hypertension	229 (50.4)	89 (40.8)	140 (59.8)	<0.001
Hypercholesterolaemia	101 (22.2)	51 (23.2)	50 (21.3)	0.53
Hypertriglyceridaemia	313 (68.9)	141 (64.1)	172 (73.5)	0.03
Mixed dyslipidaemia	173 (22)	79 (35.9)	94 (40.2)	0.35
**Laboratories**				
Elevated ALT, n (%)	158 (34.4)	62 (28.2)	96 (41)	0.001
Elevated AST, n (%)	79 (17.4)	27 (12.3)	52 (22.2)	0.004
Elevated liver enzymes, n (%)	169 (37.3)	68 (30.9)	101 (43.2)	0.001
ALT, U/L	22.9 (16.9–34.2)	20.20 (15.7–29)	25 (18.5–39.8)	<0.001
AST, U/L	20.7 (17.1–27.7)	19.4 (16.9–23.8)	22 (17.3–31)	0.002
Platelet count, 10⁹/L	245 (203–301)	255 (212–310.5)	236 (193–294.3)	0.02
HbA1c, %	8 (6.5–10.4)	7.9 (6.6–10.7)	8 (6.4–10.2)	0.63
Serum creatinine, mg/dL	0.8 (0.6–0.9)	0.7 (0.6–0.9)	0.8 (0.6–0.9)	0.02
eGFR, mL/min/m^2^	99 (83–109)	102 (86–109)	96 (81–107)	0.03
eGFR <60 mL/min/m^2^, n (%)	26 (5.7)	11 (5.1)	15 (6.5)	0.53
Triglycerides, mg/dL	137.2 (103.3–213)	121.5 (96–187.7)	160.1 (111–233.6)	<0.001
Cholesterol, mg/dL	168 (140–199)	168 (140–194.4)	170 (141–204)	0.48
LDL-c, mg/dL	91.8 (66.1–117.4)	91.7 (66.7–113.9)	91.9 (65.4–121.4)	0.48
HDL-c, mg/dL	43.8 (36–53.6)	45.4 (39–56)	42.2 (33.4–49.7)	<0.001
**FIB-4 index**				
FIB-4 index *continuous*	0.98 (0.68–1.37)	0.91 (0.66–1.28)	1.03 (0.72–1.44)	0.02
FIB-4 index *low risk*	351 (77.3)	182 (82.7)	171 (73)	0.007
FIB-4 index *intermediate risk and high risk*	103 (22.6)	38 (17.2)	65 (27.7)	

MASLD, metabolic dysfunction-associated steatotic liver disease; BMI, body mass index; CVD, cardiovascular disease; DM, diabetes mellitus; ALT, alanine aminotransferase; AST, aspartate aminotransferase; HbA1c, glycated haemoglobin; eGFR, estimated glomerular filtration rate; LDL-c, low-density lipoprotein cholesterol; HDL-c, high-density lipoprotein cholesterol. Data are expressed as medians (p25–p75), or number (percentages) when corresponds.

A total of 22.2% of patients (n = 101) met the criteria for MASH. Additionally, 22.7% (n = 103) were classified as having intermediate- or high-risk liver fibrosis according to the FIB-4 index. The proportion of patients with intermediate-to-high FIB-4 risk was significantly higher among those with MASLD compared with those without MASLD (27.7% vs. 17.2%; p = 0.007).

Among patients eligible for secondary risk assessment according to their FIB-4 score, VCTE was performed in 46 patients, and significant fibrosis (LSM ≥8.0 kPa) was observed in 12 (26%). Based on the proportion of fibrosis among patients who underwent elastography, the estimated prevalence of fibrosis among those with MASLD (n = 234) was 10.6%, while no cases were identified through the algorithmic branch of non-MASLD patients. No significant differences in age, sex, BMI, liver enzyme levels or fibrosis risk category were observed between patients eligible for transient elastography who underwent elastography and those who did not (Supplementary Table 1).

Detection-discordance analysis revealed that among 234 patients classified as having MASLD, 57% (n = 133) had normal transaminase levels despite steatosis identified by ultrasonography. Likewise, among those with intermediate- or high-risk FIB-4 scores, 43.6% (n = 45) had normal transaminase levels. This indicates that nearly half of the individuals with steatosis or increased fibrosis risk would have remained undetected if screening had relied solely on liver enzymes elevation. Similarly, only 38.6% (n = 39) of patients with MASH were correctly identified by FIB-4 as having intermediate-to-high fibrosis risk, and 37.8% (n = 39) of those with intermediate-to-high FIB-4 scores met MASH criteria, confirming their distinct and complementary usefulness in assessing liver health.

To identify factors associated with MASLD beyond descriptive group differences, we conducted univariable logistic regression analyses (Table 2), which showed that higher BMI and several routinely available biochemical laboratory measures were associated with MASLD. In the final multivariable model selected, female sex was associated with higher odds of MASLD (_a_OR 2.05, 95% CI 1.28–3.28; p < 0.001), as were elevated liver enzymes (ALT and/or AST) (_a_OR 1.93, 95% CI 1.21–3.07; p < 0.001). BMI remained independently associated with MASLD (_a_OR per 1-kg/m^2^ increase, 1.18, 95% CI 1.12–1.24; p < 0.001).

**Table 2:** Univariable and multivariable regression analysis.

Variable	Univariable OR (95% CI)	p-value	*Adjusted* OR (95%CI)	p-value
Sex, female	1.43 (0.94–2.18)	0.09	2.05 (1.28–3.28)	<0.001
Age, years	0.99 (0.98–1)	0.93		
BMI, kg/m^2^*continuous*	1.17 (1.11–1.23)	<0.001	1.18 (1.12–1.24)	<0.001
BMI, malnutrition + normal	*ref*	*ref*		
BMI, overweight	1.39 (0.79–2.46)	0.25		
BMI, obesity	5.91 (3.27–10.67)	<0.001		
BMI >35 kg/m^2^	8.45 (3.25–21.9)	<0.001		
Waist circumference	1.06 (1.04–1.08)	<0.001		
Hypertension	2 (1.35–3)	<0.001		
Elevated ALT	1.91 (1.24–2.93)	0.003		
Elevated AST	2.13 (1.21–3.74)	0.008		
Elevated liver enzymes	1.93 (1.26–2.96)	0.002	1.93 (1.21–3.07)	<0.001
Educational attainment (higher than secondary)	1.69 (1.09–2.63)	0.01		
Platelets	0.99 (0.99–0.99)	0.02		
HDL-c	0.98 (0.96–0.99)	0.02		
Hypertriglyceridaemia	1.62 (1.05–2.49)	0.02		
eGFR <60 mL/min/m^2^	1.1 (0.44–2.73)	0.82		

BMI, body mass index; ALT, alanine aminotransferase; AST, aspartate aminotransferase; HDL-c, high-density lipoprotein cholesterol; eGFR, estimated glomerular filtration rate.

**Fig. 2:**
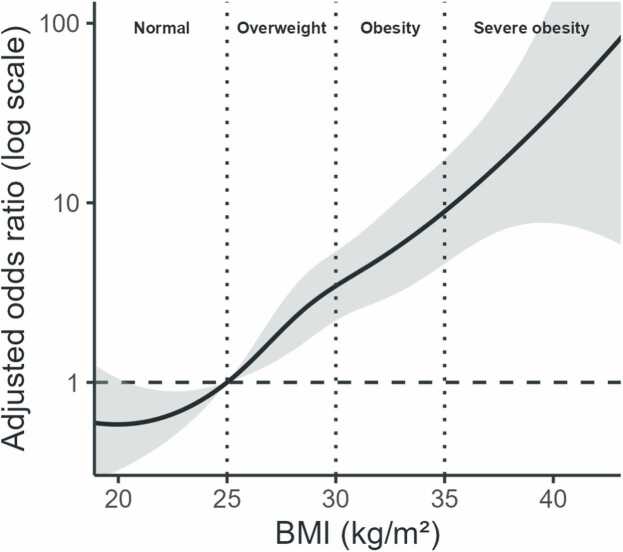
Spline-based adjusted odds ratios for MASLD across body mass index (BMI).

To further characterise the BMI–MASLD association while allowing for non-linearity, we modelled BMI as a continuous exposure using a spline-based multivariable logistic regression model and derived BMI-specific adjusted odds ratios (_a_ORs) for a 1 kg/m^2^ increase across the BMI. The per-unit association increased progressively across the normal BMI range, becoming statistically evident from approximately BMI 23–24 kg/m^2^. In the overweight range, each 1 kg/m^2^ increment was consistently associated with higher odds of MASLD. Around the transition to obesity (approximately BMI 29–31 kg/m^2^), the magnitude of the per-unit association appeared attenuated, with borderline or modest statistical support at BMI 30 (aOR 1.19, 95% CI 0.99–1.42) and evidence of association at BMI 31–34 (aORs ∼1.19–1.24). For BMI ≥35 kg/m^2^, point estimates remained above 1 and increased gradually. This finding supports a non-linear BMI–MASLD relationship, with the steepest per-unit increases in the overweight range and a less pronounced gradient around BMI 30 kg/m^2^. All estimates were adjusted for sex and elevated liver enzymes (ALT and/or AST) (Fig. 2).

## Discussion

This quality improvement initiative demonstrates the feasibility of integrating a structured diagnostic pathway for MASLD into routine diabetes care in primary healthcare settings. More than half of adults with type 2 diabetes evaluated had MASLD, and approximately one in five met criteria for MASH or showed increased risk of liver fibrosis according to the FIB-4 index. Furthermore, the cascade diagnostic approach identified an estimated 6.3% of individuals with significant liver fibrosis. These findings underscore the substantial hepatic burden associated with diabetes in the population served by the public primary care system.

Compared with the general population, people with diabetes exhibit a higher risk across all stages of the MASLD spectrum, although geographic and ethnic differences have been described.^10^ Most reported prevalences of MASLD and MASH among individuals with type 2 diabetes derive from hospital-based or population studies, averaging 65% and 31.5%, respectively,^11^ compared with 52.2% and 23.3% observed in this primary care cohort. While previous reports estimate that 35.5% of individuals with T2D and MASLD present clinically significant fibrosis, the current study found an estimated 10.6% prevalence within this category.^11^ These differences may reflect variations in healthcare setting, genetic and lifestyle background among populations and selection bias. Findings similar to ours were reported by Balkhed *et al*,^12^ who observed a prevalence of 58% for MASLD and 7% for suspected fibrosis among individuals with T2D in primary care settings.

The stepwise approach combining ultrasonography, FIB-4 risk stratification and selective VCTE enabled the identification of individuals with increased risk of liver fibrosis who would otherwise remain undetected. Detection gaps revealed that more than half of patients with steatosis or fibrosis risk had normal transaminase levels, underscoring the limitations of liver enzyme interpretation alone and the value of systematic non-invasive assessments. These findings support the operational implementation of care pathways that integrate imaging and simple fibrosis scores as part of comprehensive diabetes management.^13^ Although MASLD without fibrosis may not confer a high risk of liver-related outcomes, accumulating evidence suggests that this phenotype is associated with increased cardiovascular and renal risk, particularly in individuals with diabetes and multiple metabolic risk factors, such as the population evaluated here.^14^, ^15^ From this perspective, identification of hepatic steatosis may be clinically relevant, and evaluation of its cost-effectiveness should be considered, especially in high-risk populations.

In an exploratory secondary analysis, routinely available clinical variables were independently associated with MASLD, including female sex, elevated liver enzymes and higher BMI. Although several studies have reported higher prevalence among men, sex-related differences appear to vary with age and metabolic profile, with increasing prevalence among women after menopause.^16^, ^17^ Given that the median age of our cohort was 55 years and that women represented a larger proportion of participants in this primary care programme, the observed association may partly reflect the demographic composition of the study population. These findings may also highlight the need for further research on MASLD among women with type 2 diabetes in Latin American populations, particularly in postmenopausal age groups, where the disease burden remains relatively understudied. The non-linear association observed across BMI categories, with the steepest gradient in the overweight range and a renewed increase among individuals with severe obesity, highlights the heterogeneity of MASLD risk within the diabetes population. These findings may help inform risk stratification and prioritisation strategies within stepwise diagnostic pathways, using variables readily available in primary care settings.

This experience also generated key lessons for health system improvement. First, structured pathways for liver disease are feasible to implement within primary care workflows using existing infrastructure. Second, stepwise risk stratification optimises the use of confirmatory tools such as elastography, minimising resource demands. Finally, this approach supports both the identification of significant liver fibrosis and preventive action for liver and extrahepatic risks associated with MASLD, reinforcing comprehensive and integrated diabetes–liver care.

This study has some limitations. First, as this was a real-world quality improvement study, not all participants attended the transient elastography assessment. Therefore, a complete-case analysis restricted to verified fibrosis assessments was performed. To mitigate potential verification bias, baseline characteristics of participants who met criteria for elastography but did or did not undergo the procedure were compared to evaluate possible selection patterns. Consequently, our findings should be interpreted as reflecting outcomes among patients who completed the diagnostic work-up under real-world conditions. Second, the multivariable analysis identifying factors associated with MASLD was exploratory and based on cross-sectional data, which precludes causal inference. Finally, the study population was derived from a primary care diabetes programme in which women represented a larger proportion of participants, and therefore the findings may not fully reflect the sex distribution observed in other populations.

In conclusion, the integration of MASLD diagnostic pathway into primary care represents a critical opportunity to strengthen early detection of advanced liver disease and MASLD associated health risks among people with diabetes. Expanding these diagnostic pathways within healthcare programmes could enhance care quality, improve risk stratification and reduce downstream complications.

## CRediT authorship contribution statement

**Ana Galíndez-Fuentes:** Investigation. **Berenice Cabrera-Victoria:** Investigation. **Rubén Silva-Tinoco:** Writing – review & editing, Writing – original draft, Supervision, Project administration, Formal analysis, Conceptualization. **Erick Vladimir Martínez-de la Cruz:** Writing – review & editing, Visualization, Formal analysis. **Alejandro Avalos-Bracho:** Writing – review & editing, Supervision, Project administration. **María Fernanda Bernal-Ceballos:** Writing – review & editing, Methodology, Formal analysis. **Erick Villa-Mejía:** Writing – review & editing. **Ricardo Ulises Macías-Rodríguez:** Writing – review & editing.

## Ethics approval and consent to participate

This study was reviewed and approved by the Mexico City Ministry of Health Ethics Committee (609-010-01-18). The participants provided their written informed consent.

## Funding

This research did not receive any specific grant from funding agencies in the public, commercial or not-for-profit sectors.

## Declaration of competing interest

The authors declare that they have no known competing financial interests or personal relationships that could have appeared to influence the work reported in this paper.

## Data Availability

The data that support the findings of this study are available from the corresponding author upon reasonable request.
