# Supplementary material for: Integrating MASLD detection into diabetes care in primary care settings in Mexico: A cascade analysis
Source: Clin Med (Lond). 2026 Apr 18;26(4):100585. doi: 10.1016/j.clinme.2026.100585 (PMC13195615; doi:10.1016/j.clinme.2026.100585)
Supplement: Supplementary file 1 — Supplementary material [file mmc1.docx]

**Supplementary Table 1.** Sensitivity analysis: comparison of participants with and without VCTE assessment

| **Variable** | **Without VCTE**  **n=64** | **With VCTE**  **n=39** | **p value** |
| --- | --- | --- | --- |
| Sex, female | 42 (65.6) | 28 (71.8) | 0.66 |
| Age, years | 58.5 (53 - 63) | 58 (50.5 – 62) | 0.36 |
| BMI, kg/m^2^ | 28.4 (25.4 – 31.2) | 29.4 (25.6 – 33.2) | 0.47 |
| Waist circumference, cm | 99.5 (90.3 – 106) | 102 (93 – 113) | 0.36 |
| **BMI categories** |  |  |  |
| BMI malnutrition + normal, n (%) | 14 (21.9) | 7 (17.9) | 0.82 |
| BMI overweight, n (%) | 26 (40.6) | 18 (46.2) |  |
| BMI obese, n (%) | 24 (37.5) | 14 (35.9) |  |
| Diabetes duration, years | 13 (5 – 18) | 5 (2.5 -11) | 0.003 |
| Age at diagnosis, years | 45 (39 – 51.2) | 48 (43.5 – 56) | 0.03 |
| Age at diagnosis (<40 years), n (%) | 20 (31.2) | 6 (15.4) | 0.19 |
| Family history of DM, n (%) | 50 (78.1) | 34 (86.5) | 0.35 |
| **Comorbilities, n (%)** |  |  |  |
| Steatosis | 35 (54.7) | 29 (73) | 0.10 |
| Hypertension | 38 (59.4) | 19 (48.7) | 0.39 |
| Hypercholesterolemia | 14 (21.9) | 6 (15.4) | 0.20 |
| Hypertriglyceridemia | 5 (7.8) | 0 (0) |  |
| Mixed dyslipidemia | 11 (17.2) | 6 (15.4) |  |
| **Laboratories** |  |  |  |
| Elevated ALT, n (%) | 29 (45.3) | 21 (53.8) | 0.52 |
| Elevated AST, n (%) | 25 (39.1) | 17 (43.6) | 0.80 |
| Elevated liver enzymes, n (%) | 35 (54.7) | 23 (59) | 0.82 |
| ALT, U/L | 27 (17.3 – 47.9) | 30.9 (21.3 – 62) | 0.13 |
| AST, U/L | 26.6 (22.2 – 50.3) | 30.9 (25 – 68.9) | 0.21 |
| Platelet count, 10⁹/L | 197 (172 – 223) | 176 (156 – 196) | 0.007 |
| HbA1c, % | 7.5 (6 – 10) | 8.3 (6.9 – 10.2) | 0.06 |
| Serum cretinine, mg/dl | 0.8 (0.64 – 0.92) | 0.75 (0.66 – 0.9) | 0.37 |
| eGFR, ml/min/m^2^ | 92.2 (77.9 – 106.3) | 98.1 (86.7 – 106.7) | 0.26 |
| eGFR <60 ml/min/m^2^, n (%) | 3 (4.6) | 2 (5.1) | 0.99 |
| Triglycerides, mg/dl | 124.3 (104.2 – 181.6) | 134.1 (88 – 209.1) | 0.73 |
| Cholesterol, mg/dl | 168 (147.3 – 197.1) | 169.9 (136.6 – 195.2) | 0.71 |
| LDL-c, mg/dl | 97.8 (65.8 – 118) | 97.6 (71.8 – 118.4) | 0.69 |
| HDL-c, mg/dl | 46.7 (38.7 – 54.9) | 42.1 (35.1 – 53.2) | 0.22 |
| **FIB-4 index** |  |  |  |
| FIB-4 index *continuous* | 1.6 (1.42 – 1.93) | 2 (1.71 – 2.5) | <0.001 |
| FIB-4 index *intermediate risk* | 53 (82.8) | 31 (79.5) | 0.87 |
| FIB-4 index *high risk* | 11 (17.2) | 8 (20.5) |  |

MASLD: MASLD: metabolic dysfunction-associated steatotic liver disease; BMI: body mass index; CVD: cardiovascular disease; DM: diabetes mellitus; ALT: alanine aminotransferase; AST: aspartate aminotransferase; HbA1c: glycated haemoglobin; eGFR: estimated glomerular filtration rate; LDL-c: low-density lipoprotein cholesterol; HDL-c: high-density lipoprotein cholesterol. Data are expressed as medians (p25 – p75), or number (percentages) when corresponds.
